# Comprehensive Telehealth Model to Support Diabetes Self-Management

**DOI:** 10.1001/jamanetworkopen.2023.36876

**Published:** 2023-10-04

**Authors:** Grazia Aleppo, Robin L. Gal, Dan Raghinaru, Davida Kruger, Roy W. Beck, Richard M. Bergenstal, Terra Cushman, Korey K. Hood, Mary L. Johnson, Teresa McArthur, Amy Bradshaw, Beth A. Olson, Sean M. Oser, Tamara K. Oser, Craig Kollman, Ruth S. Weinstock

**Affiliations:** 1 Northwestern University Feinberg School of Medicine, Chicago, Illinois; 2 Jaeb Center for Health and Research, Tampa, Florida; 3 Henry Ford Health System, Detroit, Michigan; 4 International Diabetes Center, Minneapolis, Minnesota; 5 Stanford University School of Medicine, Stanford, California; 6 Cecelia Health, New York, New York; 7 Lagoon Health, Minneapolis, Minnesota; 8 University of Colorado School of Medicine, Aurora; 9 SUNY Upstate Medical University, Syracuse, New York

## Abstract

**Importance:**

As the number of patients with diabetes continues to increase in the United States, novel approaches to clinical care access should be considered to meet the care needs for this population, including support for diabetes-related technology.

**Objective:**

To evaluate a virtual clinic to facilitate comprehensive diabetes care, support continuous glucose monitoring (CGM) integration into diabetes self-management, and provide behavioral health support for diabetes-related issues.

**Design, Setting, and Participants:**

This cohort study was a prospective, single-arm, remote study involving adult participants with type 1 or type 2 diabetes who were referred through community resources. The study was conducted virtually from August 24, 2020, to May 26, 2022; analysis was conducted at the clinical coordinating center.

**Intervention:**

Training and education led by a Certified Diabetes Care and Education Specialist for CGM use through a virtual endocrinology clinic structure, which included endocrinologists and behavioral health team members.

**Main Outcomes and Measures:**

Main outcomes included CGM-measured mean glucose level, coefficient of variation, and time in range (TIR) of 70 to 180 mg/dL, time with values greater than 180 mg/dL or 250 mg/dL, and time with values less than 70 mg/dL or 54 mg/dL. Hemoglobin A_1c_ was measured at baseline and at 12 and 24 weeks.

**Results:**

Among the 234 participants, 160 had type 1 diabetes and 74 had type 2 diabetes. The mean (SD) age was 47 (14) years, 123 (53%) were female, and median diabetes duration was 20 years. Median (IQR) CGM use over 6 months was 96% (91%-98%) for participants with type 1 diabetes and 94% (85%-97%) for those with type 2 diabetes. Mean (SD) hemoglobin A_1c_ (HbA_1c_) in those with type 1 diabetes decreased from 7.8% (1.6%) at baseline to 7.1% (1.0%) at 3 months and 7.1% (1.0%) at 6 months (mean change from baseline to 6 months, −0.6%, 95% CI, −0.8% to −0.5%; *P* < .001), with an 11% mean TIR increase over 6 months (95% CI, 9% to 14%; *P* < .001). Mean HbA_1c_ in participants with type 2 diabetes decreased from 8.1% (1.7%) at baseline to 7.1% (1.0%) at 3 months and 7.1% (0.9%) at 6 months (mean change from baseline to 6 months, −1.0%; 95% CI, −1.4% to −0.7%; *P* < .001), with an 18% TIR increase over 6 months (95% CI, 13% to 24%; *P* < .001). In participants with type 1 diabetes, mean percentage of time with values less than 70 mg/dL and less than 54 mg/dL decreased over 6 months by 0.8% (95% CI, −1.2% to −0.4%; *P* = .001) and by 0.3% (95% CI, −0.5% to −0.2%, *P* < .001), respectively. In the type 2 diabetes group, hypoglycemia was rare (mean [SD] percentage of time <70 mg/dL, 0.5% [0.6%]; and <54 mg/dL, 0.07% [0.14%], over 6 months).

**Conclusions and Relevance:**

Results from this cohort study demonstrated clinical benefits associated with implementation of a comprehensive care model that included diabetes education. This model of care has potential to reach a large portion of patients with diabetes, facilitate diabetes technology adoption, and improve glucose control.

## Key Points

**Question:**

Can a virtual diabetes specialty clinic facilitate comprehensive care and support continuous glucose monitoring integration into diabetes self-management?

**Findings:**

This cohort study found that over 26 weeks, a significant reduction in mean hemoglobin A_1c_ value and a significant increase in mean time in the range 70 to 180 mg/dL were observed among patients living with type 1 and type 2 diabetes.

**Meaning:**

A virtual specialty clinic can successfully support those living with type 1 or type 2 diabetes to improve glycemic outcomes.

## Teaser

This cohort study examines glycemic outcome data for adults with type 1 or type 2 diabetes who participated in a virtual endocrinology clinic that supported continuous glucose monitoring and provided education and behavioral health support.

## Introduction

There are approximately 37.3 million individuals with diabetes in the United States, and the number continues to increase.^1^ Despite this increasing number, many patients do not have ready access to endocrinology care. In 2022, the number of endocrinologists in the United States was estimated at 8524 (a ratio of individuals with diabetes or other endocrine disorders to endocrinologist of 4375:1).^2^ The great majority of diabetes care takes place in the primary care setting.^3^

Clinical benefits of continuous glucose monitoring (CGM) have been demonstrated in type 1 and insulin-treated type 2 diabetes with a reduction in hemoglobin A_1c_ (HbA_1c_), increase in CGM-measured time in range (TIR) of 70 to 180 mg/dL, decrease in hypoglycemia incidence, and improvement in quality-of-life measurements.^4,5,6,7,8,9,10^ Despite evidence showing clinical benefits, CGM use is not yet widespread, particularly for patients whose diabetes is managed in a primary care setting.

Despite primary care clinicians’ interest in CGM use for their patients with diabetes, limited knowledge, technology readiness, and lack of resources are limiting factors in diabetes technology implementation and expansion.^11^ Navigating insurance coverage remains a key barrier, as well as access to CGM training support and education to improve understanding of data.

Telehealth offers clinicians caring for patients with diabetes an opportunity to use CGM technology to remotely monitor glucose levels and make shared decisions with patients about therapy modification without the need for an in-person visit.^12^ Evidence of the feasibility and effectiveness of remote CGM initiation through telehealth as a means of expanding access was reported in a pilot study of 34 adults with type 1 or type 2 diabetes, where reduction of HbA_1c_ value, increase in TIR, decrease in diabetes distress, and increased satisfaction with glucose monitoring were observed after 12 weeks of CGM use.^13^ Similar benefits were reported in the ONBOARD study in adults with type 1 diabetes already on CGM using a multicomponent telehealth intervention^14^ and in the Onduo Virtual Diabetes Clinic for adults with type 2 diabetes where participants were evaluated remotely by an endocrinologist and provided CGM for intermittent use without in-office training.^15^

Growing evidence of the potential for successful remote diabetes care and CGM technology use prompted this study to evaluate a model that could minimize adoption-limiting factors such as geography and access to specialty care for adults with type 1 or type 2 diabetes. The Virtual Diabetes Specialty Clinic (VDiSC) study was designed to assess the feasibility and efficacy of establishing a virtual endocrinology clinic to facilitate comprehensive diabetes care, support CGM integration into diabetes self-management, and provide behavioral health support for diabetes-related issues. Decision support technology use was also evaluated within this virtual clinic model.

## Methods

The study was conducted virtually from August 24, 2020, to May 26, 2022. Participants were recruited through an insurance company (CVS Health Clinical Trial Services) and through primary care and endocrinology clinic referrals from 2020 through 2021 and were followed up for as long as 26 weeks. Potentially eligible participants received a letter, email, and/or flyer with study information, each of which included a link to the electronic consent form. Interested individuals could ask the study team questions as part of the consent process. The protocol and informed consent form were approved by an institutional review board.

Eligible participants were US residents 18 years and older with type 1 or type 2 diabetes who used either an insulin pump or multiple daily injections of insulin. Participants were required to have a smartphone and access to a computer with internet that could be used for virtual visits. Exclusions included use of an automated insulin delivery system, pregnancy, or kidney dialysis. Participants were eligible if they were either not currently using CGM or if they were using CGM and their TIR was less than 60% or their time with values lower than 54 mg/dL was more than 1%.

### Study Procedures

Enrolled participants who were not currently using CGM completed an initial phase with a blinded Dexcom G6 Pro CGM (Dexcom) to collect baseline data. Unblinded Dexcom G6 sensors and other study supplies were shipped directly to participants for the subsequent 26 follow-up weeks. Eligible participants were assigned a Certified Diabetes Care and Education Specialist (CDCES), who served as the participant’s primary contact throughout the study. Diabetes history and demographic data including race and ethnicity were self-reported. During initial contact with the CDCES, the CDCES reviewed self-reported diabetes history data, and information about CGM was provided. Throughout the study, the CDCES provided CGM education and training and was a resource for basic diabetes management education and treatment changes.

Participants were scheduled to complete 3 remote training sessions with the CDCES; these were intended to be conducted through video. For participants new to CGM, the initial training session content covered CGM initiation, including sensor insertion, alerts and alarms, uploading data, and visualizing data. For participants who used the Dexcom G6 CGM before study enrollment, the initial training session covered CGM support as needed. The second session included training on the use of data-visualization tools and CGM data to make self-management changes in insulin dosing, meals, and exercise. The third training session included additional CGM instruction to assist participants with individualizing CGM use and provided another opportunity to troubleshoot concerns or issues. The training approach was individualized to accommodate comfort with technology and participant goals. Interim contacts with the CDCES were scheduled to encourage ongoing review of CGM data; contacts were initiated by the CDCES or by the participant on an as-needed basis, and the format of contacts included video training sessions, texts, emails, and telephone calls.

The CDCES reviewed CGM data with the participant during scheduled contacts to assist with self-management. The CDCES was authorized per study protocol to make insulin dose adjustments within a range of up to 20% for basal insulin and up to 30% for insulin boluses. A study endocrinologist was available for CDCES consultation as needed; participant referral to a study endocrinologist was available if warranted. The CDCES also had access to a decision-support app platform that generates algorithm-based recommendations for insulin dosing (insulin pump and daily injections) based on participant’s treatment regimen and needs as well as a patient-facing mobile app that includes a bolus calculator to calculate their insulin dose (endo-digital; DreaMed Diabetes). This clinical decision support was previously validated in patients with type 1 diabetes using insulin pumps and CGM,^16^ but final recommendations to participants were determined by the CDCES in consultation with the study endocrinologist when needed.

A capillary blood collection kit was sent to participants at baseline and at 12 and 26 weeks for fingerstick blood sample collection returned by mail to the Advanced Research and Diagnostic Laboratory (University of Minnesota) for HbA_1c_ measurement using the Tosch Automated Glycohemoglobin Analyzer HLC-723G8. Accuracy of this procedure for capillary blood sample collection and HbA_1c_ measurement is comparable with that of venous blood HbA_1c_ measurements.^17^

Participant self-reported outcome measures, which included the 8-item Patient Health Questionnaire, Diabetes Distress Scale (Management Distress Items), and Hypoglycemia Fear Survey worry subscale, were completed at enrollment and at 4, 8, 12, and 26 weeks. Participants who met thresholds for a positive screen on any of these 3 measures were referred for behavioral counseling and intervention. Additional questionnaires (CDC Healthy Days, Diabetes Technology Attitudes, Benefits and Burdens of CGM, Hypoglycemia Confidence Scale, Glucose Monitoring Satisfaction Survey, and a 1-item sleep survey to measure sleep quality) were also completed at enrollment, 12 weeks, and 26 weeks; results will be separately analyzed and reported.

Participants were asked monthly to report whether they experienced severe hypoglycemia (defined as an event that required assistance from another person because of altered consciousness), diabetic ketoacidosis, and hospitalizations. Available CGM glucose values were assessed to confirm self-reported severe hypoglycemia events, and the medical monitor reviewed the self-reported event description.

### Outcome Measures

Efficacy outcomes included CGM use; CGM metrics for hypoglycemia (<54 and <70 mg/dL), hyperglycemia (>180 and >250 mg/dL), and TIR for 70 to 180 mg/dL; mean glucose level, and glycemic variability (coefficient of variation), HbA_1c_ value, and participant-reported outcomes, including psychosocial and diabetes treatment satisfaction questionnaires. Safety outcomes included severe hypoglycemia, diabetic ketoacidosis, hospitalizations, and emergency department visits.

### Statistical Analysis

To be included in the analyses, it was necessary for a participant to initiate real-time (unblinded) CGM use, complete at least 1 training session, and have at least 168 hours of CGM data during follow-up. A linear mixed model with a random-participant effect was used to test mean change from baseline to follow-up. For metrics with a skewed distribution, robust means were calculated using an M-estimator to down-weight outliers. Descriptive statistics included means with SDs or medians with IQRs depending on the distribution of data. Missing HbA_1c_ values were imputed using the Glucose Management Indicator (GMI) formula^18^ to convert CGM-measured mean glucose into an HbA_1c_ equivalent (n = 43 at 6 months). Missing HbA_1c_ values without a corresponding CGM mean glucose available (n = 8 at 6 months) and questionnaire values at baseline, 3 months, and 6 months were handled using direct likelihood in the mixed model. For the glycemic outcomes, 2 participants missing baseline CGM data were excluded from the pre/post comparison. A mixed model was used to assess potential risk factors for the change in HbA_1c_ value from baseline to 6 months. The false discovery rate was used to adjust for multiple comparisons. *P* < .05 was used to define statistical significance. Analyses were performed separately by diabetes type using SAS version 9.4 (SAS Institute).

## Results

Among the 234 participants included in the analyses, 160 had type 1 diabetes and 74 had type 2 diabetes. The mean (SD) age was 47 (14) years, 123 (53%) were female, and median diabetes duration was 20 years. The 234 participants included in the analyses were a subset of the 341 who signed the study consent and were screened; 75 were ineligible, 31 dropped out before initiating the study, and 1 dropped out immediately after the CDCES sessions without obtaining any CGM data (eFigure 1 in Supplement 1).

### Participants With Type 1 Diabetes

The mean (SD) age of the 160 participants was 44 (14) years, 92 (58%) were female, and median diabetes duration was 23 years. A total of 72 participants (45%) used an insulin pump, while 88 (55%) used multiple daily injections of insulin; 47 (29%) were already using CGM at enrollment (Table 1).

**Table 1.** Participant Demographic Data and Clinical Characteristics at Enrollment (N = 234)

Characteristic	No. (%)	
	Type 1 diabetes (n = 160)	Type 2 diabetes (n = 74)
Currently use CGM	47 (29)	0
Age, mean (SD), y	44 (14)	55 (12)
<25	9 (6)	1 (1)
25 to <40	62 (39)	9 (12)
40 to <50	33 (21)	13 (18)
50 to <65	41 (26)	39 (53)
≥65	15 (9)	12 (16)
Gender		
Female	92 (58)	31 (42)
Male	68 (42)	43 (58)
Race		
American Indian or Alaskan Native	1 (<1)	0
Asian	4 (3)	4 (5)
Black or African American	10 (6)	10 (14)
White	132 (83)	55 (74)
Other^a^	8 (5)	3 (4)
Do not wish to answer	5 (3)	2 (3)
Hispanic ethnicity		
Hispanic or Latino	9 (6)	4 (5)
Not Hispanic or Latino	147 (92)	67 (91)
Do not wish to answer	3 (2)	0
Do not know	1 (<1)	3 (4)
Body mass index, median (IQR)^b^	27 (23-32)	35 (30-40)
Diabetes duration, median (IQR), y	23 (13-34)	19 (12-24)
Range	<1-72	<1-69
Baseline HbA_1c_, mean (SD), %^c^	7.8 (1.6)	8.1 (1.7)
<7.0%	55 (34)	23 (31)
7.0% to 7.4%	21 (13)	6 (8)
7.5% to 8.9%	54 (34)	22 (30)
≥9.0%	30 (19)	23 (31)
Insulin modality		
Multiple daily injections (basal-bolus therapy)	88 (55)	73 (99)
Pump	72 (45)	1 (1)
Diabetes health care professional		
Endocrinologist	117 (73)	43 (58)
Primary care clinician	43 (27)	31 (42)
Health insurance coverage		
Private (includes ACA and single service plans)	124 (78)	56 (76)
Government sponsored	29 (18)	17 (23)
No insurance	3 (2)	1 (1)
Do not know/do not wish to answer	4 (3)	0
Annual household income, $		
<50 000	44 (28)	24 (32)
50 000-<100 000	56 (35)	29 (39)
≥100 000	36 (23)	15 (20)
Do not know/do not wish to answer	24 (15)	6 (8)

Abbreviations: ACA, Affordable Care Act; CGM, continuous glucose monitoring; HbA_1c_, hemoglobin A_1c_.

^a^ Other self-identified race: Black and White (n = 3); Asian and White (n = 2); Native American and White (n = 1); White, Black, and Hispanic (n = 1); and White and Jewish (n = 1) among participants with type 1 diabetes and Mexican and White (n = 1); Black, White, and unknown (n = 1); and American Indian and White (n = 1) among those with type 2 diabetes.

^b^ Calculated as weight in kilograms divided by height in meters squared.

^c^ Values provided by central laboratory. Two HbA_1c_ values were missing and were replaced with self-reported values within the last 6 months. Two additional HbA_1c_ laboratory values were missing with no self-reported value within the last 6 months; values were imputed using the glycemic mean at baseline with the glucose management indicator formula.^18^

Median (IQR) CGM usage over 6 months was 96% (91%-98%), consistent between the first and the second 3-month follow-up periods. This was consistent among prior CGM users (93%; n = 47) and non-CGM users (97%; n = 113). Mean (SD) HbA_1c_ decreased from 7.8% (1.6%) at baseline to 7.1% (1.0%) at 3 months and 7.1% (1.0%) at 6 months (mean change from baseline to 6 months, −0.6%, 95% CI, −0.8% to −0.5%; *P* < .001) (Table 2, Figure, A, and eFigure 2A in Supplement 1). These improvements were consistent across levels of diabetes type, prior CGM use, diabetes duration, presence of a chronic health condition, self-reported amount of exercise, and healthy eating (eTable 1 in Supplement 1).

**Table 2.** Glycemic Results by Diabetes Type

Result	Baseline, No. (%)	Months 1-6 of follow-up	Mean change (95% CI), %	*P* value for follow-up vs baseline^a^
**Type 1 diabetes**				
CGM data, No.	158^b^	160	NA	NA
Hours of CGM data, median (IQR)	237 (232 to 336)	4175 (3955 to 4241)	NA	NA
Glucose, mean (SD), mg/dL	183 (49)	165 (30)	−18 (−23 to −13)	<.001
% of Time with values, mean (SD)				
TIR (70-180 mg/dL)	50 (20)	61 (16)	11 (9 to 14)	<.001
>180 mg/dL	45 (24)	36 (17)	−10 (−12 to −8)	<.001
>250 mg/dL^c^	19 (16)	11 (11)	−8 (−10 to −6)	<.001
>300 mg/dL^c^	8.7 (9.3)	4.3 (6.0)	−4.5 (−5.6 to −3.5)	<.001
<70 mg/dL^c^	3.8 (4.8)	2.9 (3.4)	−0.8 (−1.2 to −0.4)	.001
<54 mg/dL^c^	0.9 (1.6)	0.5 (0.9)	−0.3 (−0.5 to −0.2)	<.001
Coefficient of variation, mean (SD)^c^	37 (7)	36 (5)	−1 (−2 to −0.4)	.004
In target range >70% of time^d^	23 (15)	49 (31)	16 (11 to 23)	<.001
In target range >70% and <70 mg/dL <4% of time^d^	6 (4)	26 (16)	13 (7 to 18)	.007
HbA_1c_, No.^b^	160	156	NA	NA
Mean (SD), %	7.8 (1.6)	7.1 (1.0)	−0.6 (−0.8 to −0.5)	<.001
**Type 2 diabetes**				
CGM data, No.	74	74	NA	NA
Hours of CGM data, median (IQR)	236 (223 to 238)	4054 (3703 to 4213)	NA	NA
Glucose, mean (SD), mg/dL	199 (58)	166 (29)	−33 (−43 to −23)	<.001
% of Time with values, mean (SD)				
TIR (70-180 mg/dL)	48 (32)	66 (20)	18 (13 to 24)	<.001
>180 mg/dL	51 (32)	33 (21)	−18 (−24 to −13)	<.001
>250 mg/dL^c^	22 (23)	8 (10)	−13 (−18 to −9)	<.001
>300 mg/dL^c^	7.1 (10.8)	2.5 (4.4)	−4.5 (−6.4 to −2.7)	<.001
<70 mg/dL^c^	0.3 (0.7)	0.5 (0.6)	0.1 (0.0 to 0.2)	.05
<54 mg/dL^c^	0.05 (0.13)	0.07 (0.14)	0.03 (0.00 to 0.04)	.006
Coefficient of variation, mean (SD)^c^	27 (7)	28 (5)	1.6 (0.3 to 3.0)	.02
In target range >70% of time^d^	21 (28)	38 (51)	23 (14 to 34)	<.001
In target range >70% and <70 mg/dL <4% of time^d^	18 (24)	37 (50)	26 (15 to 36)	<.001
HbA_1c_, No.^e^	74	70	NA	NA
Mean (SD), %	8.1 (1.7)	7.1 (0.9)	−1.0 (−1.4 to −0.7)	<.001

Abbreviations: CGM, continuous glucose monitoring; HbA_1c_, hemoglobin A_1c_; NA, not applicable; TIR, time in range.

^a^ *P* values were adjusted for multiple comparisons using the 2-stage Benjamini-Hochberg adaptive false discovery rate procedure.

^b^ Two participants were missing baseline CGM data.

^c^ For metrics with a skewed distribution, robust means using an M-estimator were calculated to down-weight outliers.

^d^ Target range was 70 to 180 mg/dL.

^e^ The model includes all available data, imputations were done when mean CGM glucose values during the previous month were available using the glucose management indicator formula,^18^ still missing data were handled using direct-likelihood method, and *P* value is for 6-month values vs baseline.

**Figure.**
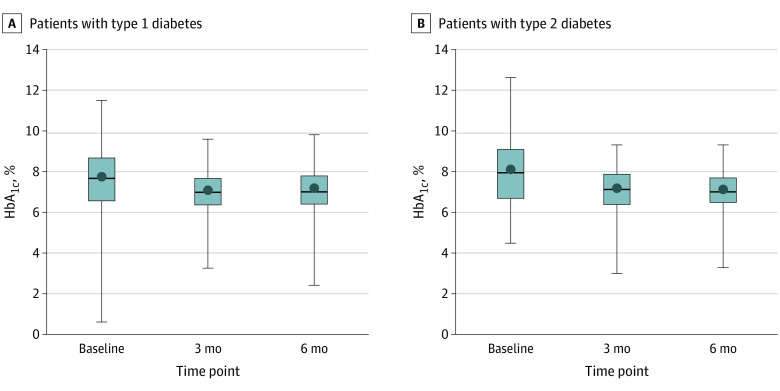
Boxplots of Hemoglobin A_1c_ (HbA_1c_) Values for Study Participants With 6 Months of Follow-Up Data shown represent 156 participants with type 1 diabetes (A) and 70 with type 2 diabetes (B). Dots denote mean values; horizontal lines inside the boxes, medians; bottom and top borders of the boxes, IQR; whiskers, range of values.

Mean (SD) TIR increased from 50% (20%) at baseline to 61% (16%) over 6 months (mean change, 11%; 95% CI, 9% to 14%; *P* < .001) (eFigure 3A in Supplement 1). Other CGM metrics reflective of hyperglycemia showed similar improvement. Improvement was observed among both prestudy CGM users and those not using CGM at study enrollment (eTable 2 in Supplement 1). Mean (SD) percentage of time below 54 mg/dL was 0.9% (1.6%) at baseline and 0.5% (0.9%) at follow-up (mean change, −0.3%; 95% CI, −0.5% to −0.2%; *P* < .001). Similarly, mean (SD) percentage of time below 70 mg/dL was 3.8% (4.8%) at baseline and 2.9% (3.4%) at follow-up (mean change, −0.8%; 95% CI, −1.2% to −0.4%; *P* = .001).

Participants completed up to 3 planned training sessions. The median (IQR) number of additional follow-up contacts per participant was 8 (6-10). Follow-up contact formats included video training sessions (80%), telephone calls (9%), texts (6%), or emails (5%). There were 89 participants (56%) with at least 1 mental health visit; 82 (92%) had an initial visit following a positive alert on a screening questionnaire, while 7 were self-referred. Overall, there were 298 mental health visits.

On the 6-month questionnaire, nearly all participants (146 of 147) reported that CGM use helped them manage their diabetes better. A majority either strongly agreed (71%) or agreed (22%) that working with the virtual clinic helped with diabetes management (eTable 3 in Supplement 1). Participant-reported outcomes showed a decrease in Diabetes Distress Scale score (mean [SD] change from baseline, −0.3 [0.6]; *P* < .001) as well as a reduction on the Hypoglycemia Fear Survey worry subscale (mean [SD] change from baseline, −2.9 [4.2]; *P* < .001) from baseline to 6 months (eTable 4 in Supplement 1).

Twenty-three participants (14%) self-reported a total of 30 adverse events, including 16 severe hypoglycemia events in 15 participants (9%), 1 of whom was diagnosed with COVID-19 and subsequently hospitalized, and 13 other hospitalizations in 10 participants (6%). As hospitalizations were self-reported, lack of hospital records precluded the ability to determine if any hospitalizations also represented diabetic ketoacidosis. The rates of severe hypoglycemia and hospitalization events were 18 and 15 events per 100 person-years, respectively.

### Participants With Type 2 Diabetes

The mean (SD) age of the 74 participants was 55 (12) years, 31 (42%) were female, and median diabetes duration was 19 years. All but 1 participant used multiple daily injections of insulin, and none were using CGM at enrollment.

Median (IQR) CGM usage over 6 months was 94% (85%-97%), consistent between the first and second 3 months. Mean (SD) HbA_1c_ decreased from 8.1% (1.7%) at baseline to 7.1% (1.0%) at 3 months and 7.1% (0.9%) at 6 months (mean change from baseline to 6 months, −1.0%; 95% CI, −1.4% to −0.7%; *P* < .001) (Table 2, Figure, B, and eFigure 2B in Supplement 1). Mean (SD) TIR was 48% (32%) at baseline and 66% (20%) at follow-up (mean change, 18%; 95% CI, 13% to 24%; *P* < .001) (eFigure 3B in Supplement 1). Mean (SD) glucose was 199 mg/dL (58 mg/dL) at baseline and 166 mg/dL (29 mg/dL) at follow-up (mean change, −33 mg/dL; 95% CI, −43 to −23 mg/dL; *P* < .001). CGM values below 54 mg/dL were rare at both times with a mean (SD) of 0.05% (0.13%) at baseline and 0.07% (0.14%) at follow-up (mean change, 0.03%; 95% CI, <0.01% to 0.04%; *P* = .006). Mean (SD) percentage of time below 70 mg/dL was 0.3% (0.7%) at baseline and 0.5% (0.6%) at follow-up (mean change, 0.1%; 95% CI, 0.0% to 0.2%; *P* = .05).

The median (IQR) number of follow-up contacts per participant after completion of the 3 remote training sessions was 8 (6-11). Follow-up contact formats included video training sessions (74%), telephone calls (13%), emails (8%), and texts (5%). There were 32 participants (43%) with at least 1 mental health visit; all were referred for an initial visit following a positive alert on a screening questionnaire. Overall, there were 74 mental health visits.

All 61 participants who responded to the 6-month questionnaire reported that CGM use helped them manage their diabetes better and either strongly agreed (85%) or agreed (15%) that working with the virtual clinic helped with diabetes management (eTable 3 in Supplement 1). Participant-reported outcomes showed a decrease in Diabetes Distress Scale score (mean [SD] change from baseline, −0.8 [0.9]; *P* < .001) as well as a reduction on the Hypoglycemia Fear Survey worry subscale (mean [SD] change from baseline, −2.8 [6.2]; *P* = .02) at 6 months (eTable 4 in Supplement 1).

Thirteen participants (18%) self-reported a total of 20 adverse events: 1 severe hypoglycemia event in 1 participant (1%) and 19 hospitalizations in 12 participants (16%). The rate of severe hypoglycemia and hospitalization were 2 and 46 events per 100 person-years, respectively.

## Discussion

Care for patients with diabetes is affected by the low number of endocrinologists compared with the growing number of patients with diabetes and often limited access to diabetes education in primary care and even in endocrinologists’ practices. This may prevent patients with diabetes from adopting devices with established clinical benefits such as CGM. Primary care clinicians frequently lack resources to implement use of diabetes technology. In a cross-sectional web-based survey to assess CGM prescribing behaviors, Oser et al^11^ noted that the most needed resources to support CGM in primary care were CGM training and workshops, education, or consultations on insurance coverage.

The VDiSC study was designed before the COVID-19 pandemic to address challenges of access to care through a telehealth care model that could be individualized to include education, technology, and behavioral health support. The 6-month follow-up results of this prospective study showed substantial reduction of HbA_1c_ values and improvement in TIR.

Several models of virtual diabetes care have been developed and described in the last few years, in part related to the COVID-19 pandemic creating the need to establish remote management options for chronic diseases. Successful remote management of type 1 diabetes, which in 52% of the patients included CGM initiation, was described by Eilan and Drincic,^19^ where endocrinologist-based telehealth visits to rural clinics showed decreases in HbA_1c_ values. Even though this model showed success in improving type 1 diabetes outcomes, it still required a rural clinic location where individuals were remotely evaluated and treated.

Another virtual model, the Onduo Virtual Diabetes Clinic, is designed to support diabetes management in the primary care setting through interim endocrinology visits for individuals with type 2 diabetes.^15,20,21^ Features include a mobile app that connects wirelessly to glucose meters and CGM devices and allows for medication tracking, remote lifestyle coaching, and clinical support. Preliminary data for 740 participants suggested improvement in HbA_1c_ values.^20^ Similarly, a subsequent prospective single-arm study with 55 participants with type 2 diabetes and intermittent CGM use over 4 months also showed a decrease in HbA_1c_ values of 1.2% from a baseline of 8.9% and an increase in TIR of 10.2% from a baseline of 65.4%.^21^

While intermittent use of CGM has been shown to have benefits,^22^ daily use of CGM provides the greatest benefits.^10,23^ Another virtual diabetes care model, Steady Health,^24^ incorporates CGM and a multidisciplinary approach with person-centered diabetes care. Their real-world retrospective case series explored early glycemic outcomes of participants with TIR less than 70% from baseline CGM data. Fifty-three participants met criteria for analysis, and an increase in median (IQR) TIR of 16.6% (6.0%-27.9%) was observed when comparing the last available 4 weeks of CGM data to baseline (mean duration of care, 11 months). There was an HbA_1c_ reduction of 1.2% in the 27 participants with data for both baseline and follow-up HbA_1c_. While these data lend support for the benefits of potential virtual diabetes care, data were retrospective, and all participants were already on CGM when they entered the study.

Although the VDiSC study included a CGM intervention, it was combined with comprehensive diabetes education, care, and behavioral support, all of which were done concomitantly and personalized based on the patient’s needs. Of note, even though the majority of patients with type 1 diabetes and approximately half with type 2 diabetes in our study reported that they received care through an endocrinologist, several factors could have prevented them from successfully adopting technology and achieving their glycemic goals. With limited access to endocrinology care, long waiting time between appointments, unavailable flexible schedules for working patients, and the long travel distance for many patients, optimization of treatment plans for these participants might have been limited even in the setting of endocrine care. In fact, these challenges may not have been overcome without the virtual intervention of intensive, on-demand diabetes education and behavioral support, none of which is readily available in endocrinology practices, let alone primary care settings.

In addition to the clinical benefits of HbA_1c_ reduction and improvement in CGM glucometrics, participant CGM experience was positive. Our study showed that 99% of the participants felt CGM use helped them better manage their diabetes regardless of diabetes type. Similarly, participants relied on real-time alerts and trends on their CGM display device more than 92% of the time; 71% of participants with type 1 diabetes and 95% with type 2 diabetes stated that CGM helped them change eating habits. Our results are consistent with published literature where the use of intermittent or short-term CGM in a virtual diabetes management model showed high CGM satisfaction scores, and 94.7% of 594 individuals with type 2 diabetes agreed that they were comfortable inserting the sensor remotely and that CGM improved their understanding of the impact of eating.^15^ Furthermore, in focus groups with parents of youth with type 1 diabetes who initiated CGM over telehealth within 30 days of diagnosis during the pandemic, the majority preferred the virtual format, describing multiple benefits from virtual visits, including convenient access to high-quality care from the comfort of home.^25^

### Strengths and Limitations

Strengths of the study included a telemedicine care approach with education and behavioral health support that could be implemented broadly by a health care plan or another organization. Participants had access to endocrinologists through the virtual clinic if a specific situation warranted escalation of care. Participant retention was high because of the flexibility of a telehealth structure, which was able to support individual participant needs and minimize burden and stress that can be associated with traditional care structures.

This study has several limitations. The study cohort was predominantly White with private insurance, so the study may not fully address the potential challenges of individuals with decreased access to care in general due to health disparities. The majority of participants with type 1 diabetes received diabetes care from endocrinologists rather than primary care. Inclusion criteria required that participants have either an Android or iOS smartphone to provide the virtual clinic team with consistent access to CGM data without needing to upload a receiver. Access to a compatible computer with internet was also required. These requirements may have affected study participation. However, the use of cellular phones has dramatically increased, and as of 2021, 85% of the US population owns a smartphone without differences according to race and ethnicity.^26^ The follow-up period could be considered relatively short at 6 months, so it will be important to evaluate whether the observed benefits can be sustained over a longer period. Results from a 6-month extension study will address this question. Finally, the CGM choice was limited to 1 brand, and it is unknown whether using other commercially available CGM brands would affect the results.

## Conclusions

The VDiSC study found that patients with diabetes experienced clinical benefits associated with the implementation of a virtual clinic care model, as demonstrated through measurable glycemic outcomes and CGM metrics, that also offered expanded specialty care for diabetes-related issues. Although it is always recommended that patients with diabetes have annual in-person evaluations with their primary clinicians to assess possible long-term complications, such as foot and eye examinations, this virtual model has the potential to expand access to care, reducing health disparities for patients unable to access diabetes care in person, and to facilitate the adoption of technologies and support targeted diabetes self-management education. This study model was designed before the COVID-19 pandemic, but the results demonstrated how diabetes telehealth services, made more readily available during the pandemic, can be successful and should be allowed to continue. Longer-duration studies and cost analyses are needed to support expansion of virtual care models.

## Notes

Supplement 1. **eFigure 1.** Flowchart of Screened, Initiated CGM, and Analyzed Participants **eFigure 2.** Scatter Plot of Baseline vs 6 Month HbA1c **eFigure 3.** Scatter Plot of Baseline vs 6 Month % time in range 70-180 mg/dL (TIR) **eTable 1.** HbA1c at Baseline, 6 Months, and Change From Baseline to 6 Months by Selected Baseline Factors **eTable 2.** Among T1D, Glycemic Results by CGM Use at Enrollment **eTable 3.** 6-Month Participant-Reported Questionnaire **eTable 4.** Participant-Reported Outcomes by Diabetes Type

Supplement 2.
Data Sharing Statement
