# Supplementary material for: Comprehensive Telehealth Model to Support Diabetes Self-Management
Source: JAMA Netw Open. 2023 Oct 4;6(10):e2336876. doi: 10.1001/jamanetworkopen.2023.36876 (PMC10551767; doi:10.1001/jamanetworkopen.2023.36876)
Supplement: Supplement 1. — eFigure 1. Flowchart of Screened, Initiated CGM, and Analyzed Participants eFigure 2. Scatter Plot of Baseline vs 6 Month HbA1c eFigure 3. Scatter Plot of Baseline vs 6 Month % time in range 70-180 mg/dL (TIR) eTable 1. HbA1c at Baseline, 6 Months, and Change From Baseline to 6 Months by Selected Baseline Factors eTable 2. Among T1D, Glycemic Results by CGM Use at Enrollment eTable 3. 6-Month Participant-Reported Questionnaire eTable 4. Participant-Reported Outcomes by Diabetes Type [file jamanetwopen-e2336876-s001.pdf]

Supplemental Online Content

Aleppo G, Gal RL, Raghinaru D, et al. Comprehensive telehealth model to support diabetes self-management. *JAMA Netw Open*. 2023;6(10):e2336876.
doi:10.1001/jamanetworkopen.2023.36876

eFigure 1. Flowchart of Screened, Initiated CGM, and Analyzed Participants
eFigure 2. Scatter plot of Baseline versus 6 Month HbA1c
eFigure 3. Scatter plot of Baseline versus 6 Month % time in range 70-180 mg/dL (TIR)
eTable 1. HbA1c at Baseline, 6 Months, and Change from Baseline to 6 Months by Selected Baseline Factors
eTable 2. Among T1D, Glycemic Results by CGM Use at Enrollment
eTable 3. 6-Month Participant-Reported Questionnaire
eTable 4. Participant-Reported Outcomes by Diabetes Type

This supplemental material has been provided by the authors to give readers additional information about their work.

© 2023 Aleppo G et al. *JAMA Network Open.*

eFigure 1. Flowchart of Screened, Initiated CGM, and Analyzed Participants

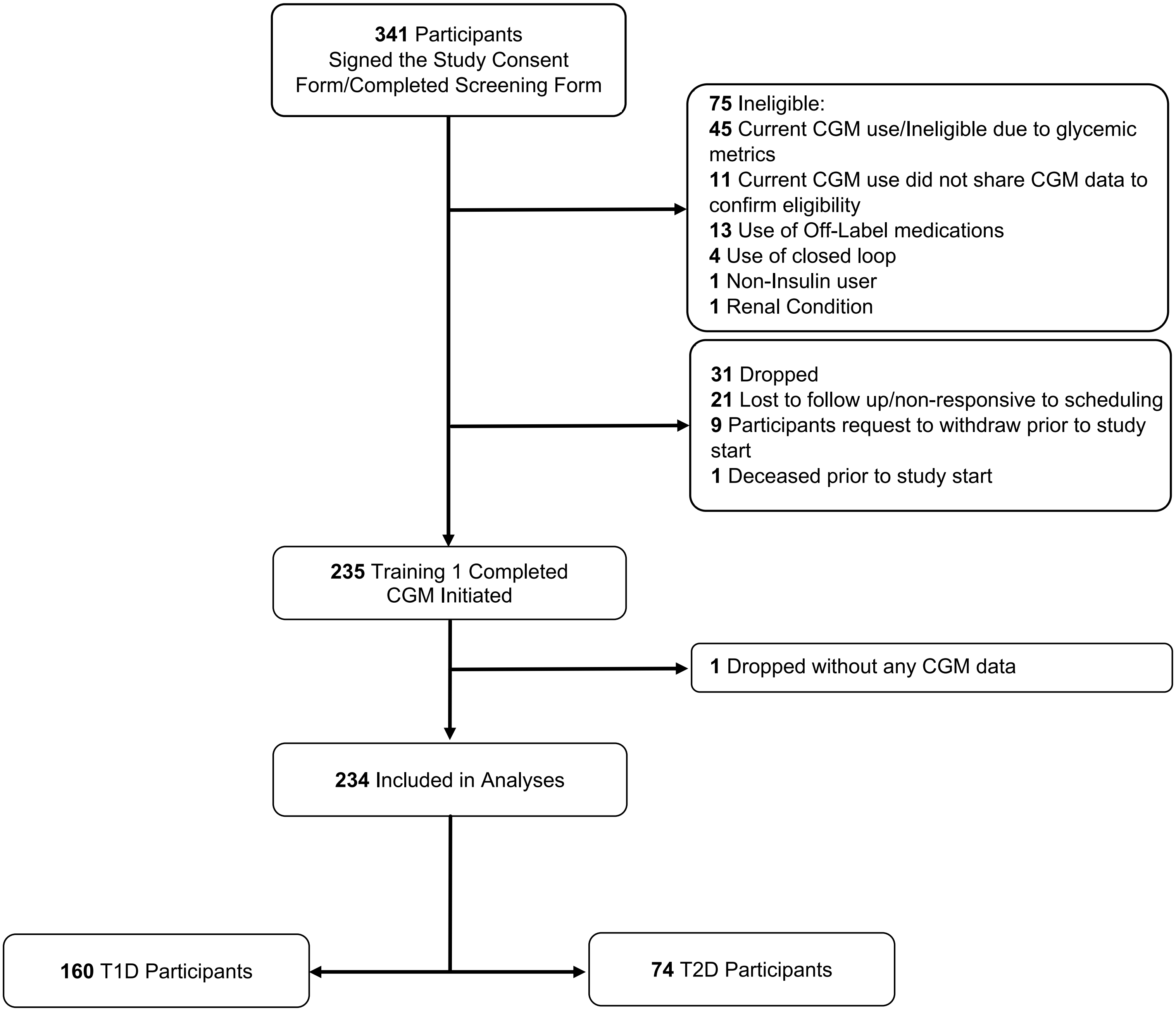

© 2023 Aleppo G et al. *JAMA Network Open.*

eFigure 2. Scatter plot of Baseline versus 6 Month HbA1c – N=156 participants with T1D in panel A and N=70 participants with T2D in panel B. The points below the diagonal line represent participants who had a better HbA1c at 6 Months when compared with Baseline.

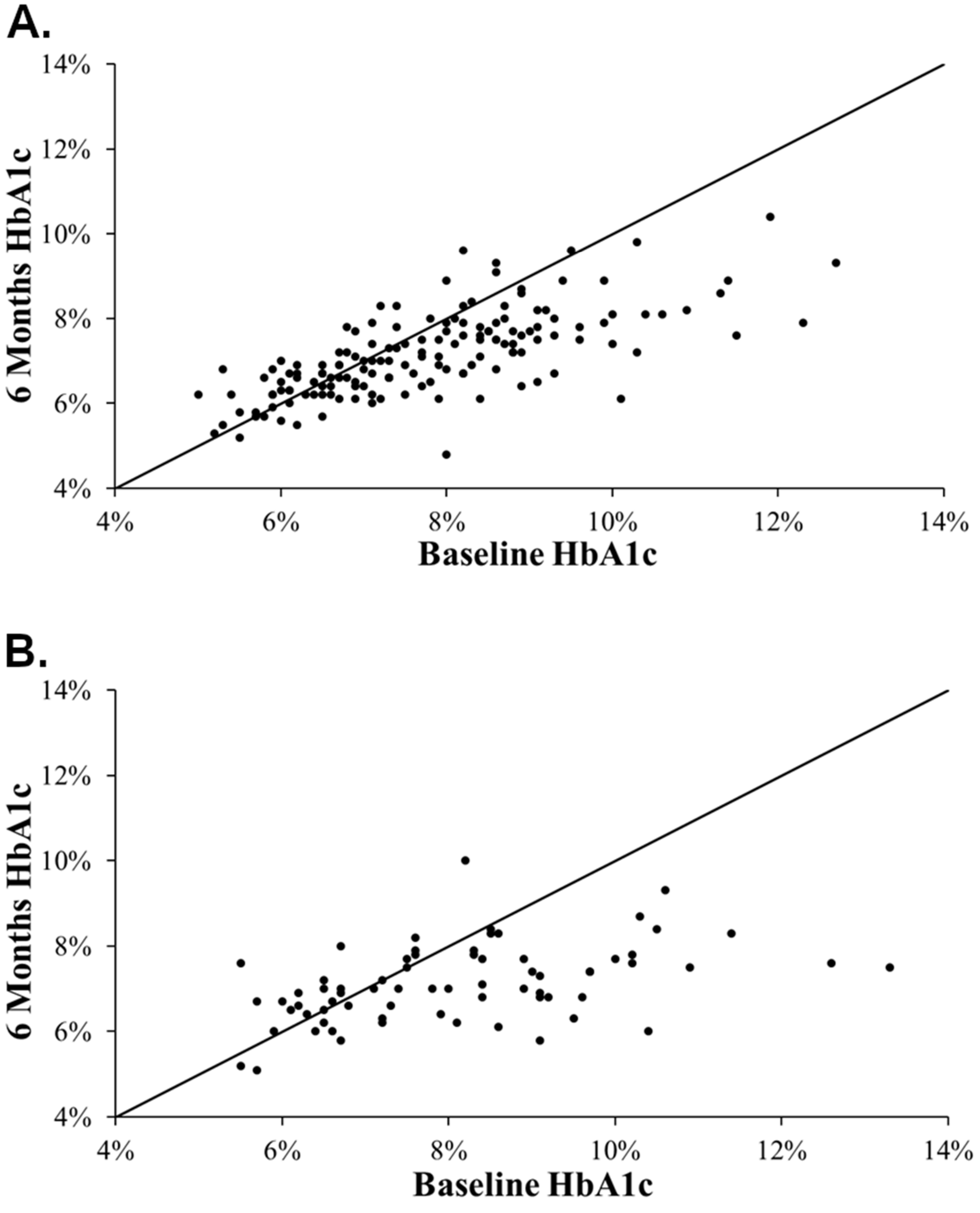

© 2023 Aleppo G et al. *JAMA Network Open.*

eFigure 3. Scatter plot of Baseline versus 6 Month % time in range 70-180 mg/dL (TIR) – N=158 participants with T1D in panel A and N=74 participants with T2D in panel B. The points above the diagonal line represent participants who had a better % time in range 70-180 mg/dL over 6 Months when compared with Baseline.

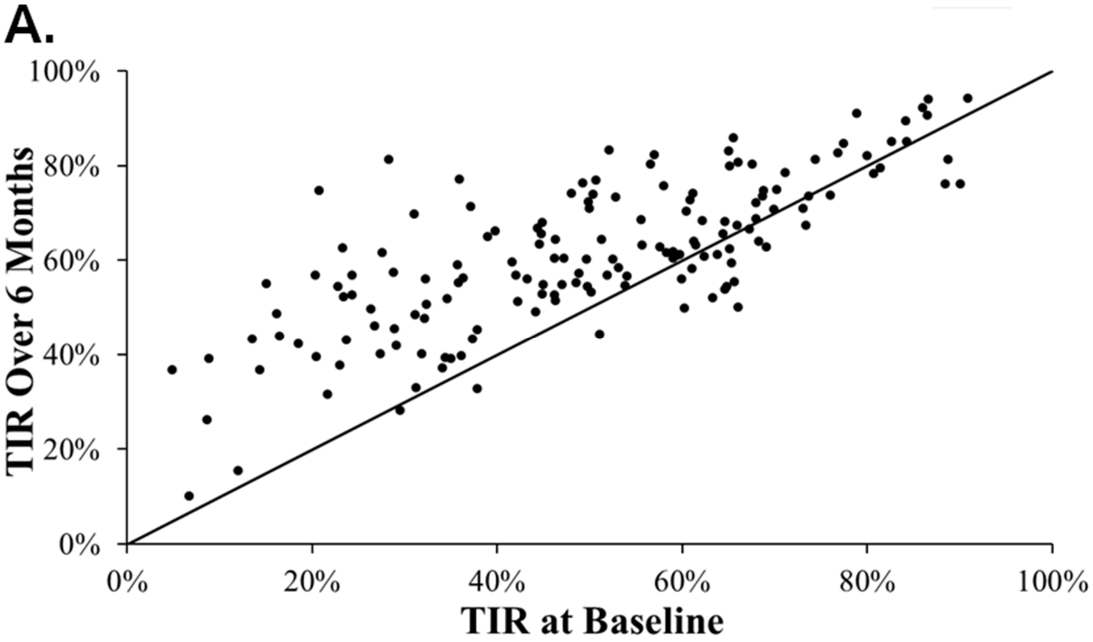

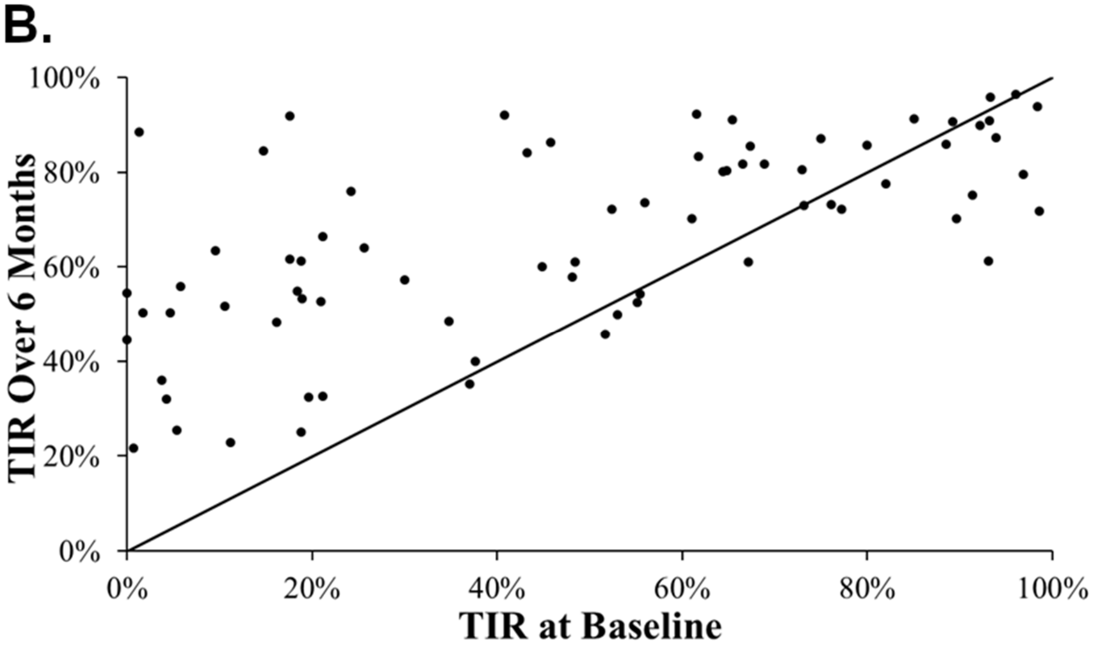

© 2023 Aleppo G et al. *JAMA Network Open.*

eTable 1. HbA1c at Baseline, 6 Months, and Change from Baseline to 6 Months by Selected Baseline Factors – Among Participants with HbA1c Values at Baseline and 6 Months (N=226)

	N	Baseline	6 Months	Change from Baseline to 6 Months	P-Value for the Change from Baseline to 6 Months[a]
**All**	226	7.9% (1.6%)	7.1% (1.0%)	-0.7% (1.3%)	
**Diabetes**					0.41
T1D	156	7.8% (1.6%)	7.1% (1.0%)	-0.6% (1.1%)	
T2D	70	8.1% (1.7%)	7.1% (0.9%)	-1.0% (1.5%)	
**Current CGM Use**					0.41
No	180	7.9% (1.6%)	7.1% (0.9%)	-0.8% (1.3%)	
Yes	46	7.7% (1.6%)	7.3% (1.2%)	-0.5% (1.2%)	
**Diabetes Duration**					0.41
<20yrs	103	8.0% (1.6%)	7.2% (1.0%)	-0.8% (1.3%)	
≥20yrs	123	7.8% (1.6%)	7.1% (1.0%)	-0.7% (1.2%)	
**Chronic health conditions other than diabetes**					0.93
No	131	7.8% (1.5%)	7.1% (1.0%)	-0.7% (1.2%)	
Yes	95	7.9% (1.7%)	7.2% (1.0%)	-0.8% (1.4%)	
**Indicated goal to improve diabetes management by healthy eating**					0.41
Yes	113	8.1% (1.8%)	7.2% (1.0%)	-0.9% (1.4%)	
No	113	7.6% (1.4%)	7.1% (1.0%)	-0.6% (1.0%)	
**Days per week spent doing at least 30 minutes of exercise**					0.41
0	31	8.0% (1.7%)	7.2% (1.0%)	-0.8% (1.2%)	
1	19	8.5% (1.5%)	7.1% (0.9%)	-1.4% (1.2%)	
2	27	7.4% (1.5%)	6.9% (0.9%)	-0.5% (1.0%)	
3	38	7.7% (1.4%)	7.0% (1.0%)	-0.7% (1.1%)	
4	28	8.3% (1.9%)	7.3% (1.1%)	-1.1% (1.6%)	
5	29	7.7% (1.4%)	7.1% (0.8%)	-0.6% (1.0%)	
6	16	8.0% (1.8%)	7.2% (1.1%)	-0.9% (1.8%)	
7	26	7.5% (1.4%)	7.2% (1.2%)	-0.3% (1.2%)	
No answer/Don’t know	12	8.0% (1.9%)	7.4% (1.1%)	-0.6% (1.1%)	

[a]P-values were adjusted for multiple comparisons using the Two Stage Benjamini-Hochberg adaptive false discovery rate procedure. Note that the FDR procedure resulted in a common p-value for different comparisons.

© 2023 Aleppo G et al. *JAMA Network Open.*

eTable 2. Among T1D[a], Glycemic Results by CGM Use at Enrollment

	**Baseline**	**Months 1-6 of Follow-Up**
**Previous CGM User**	N=46	N=47
Hours of CGM Data - *Median (IQR)*	642 (481, 674)	4038 (3816, 4211)
Mean Glucose (mg/dL) - *Mean (SD)*	176 (53)	170 (38)
% Time in Target Range (70-180 mg/dL) - *Mean (SD)*	52% (20%)	59% (19%)
% Time >180 mg/dL - *Mean (SD)*	42% (26%)	38% (21%)
% Time >250 mg/dL – *Mean (SD)* [b]	18.1% (17.2%)	14.2% (13.9%)
% Time >300 mg/dL – *Mean (SD)* [b]	8.7% (9.3%)	6.1% (8.3%)
% Time <70 mg/dL – *Mean (SD)* [b]	4.2% (5.2%)	3.1% (3.3%)
% Time <54 mg/dL – *Mean (SD)* [b]	1.06% (1.60%)	0.65% (0.86%)
Coefficient of Variation – *Mean (SD)* [b]	37% (6%)	36% (5%)
HbA1c - *N*	47	46
Mean (SD)	7.7% (1.6%)	7.3% (1.2%)
**Non-CGM User**	N=112	N=113
Hours of CGM Data - *Median (IQR)*	236 (230, 238)	4197 (4039, 4247)
Mean Glucose (mg/dL) - *Mean (SD)*	186 (48)	164 (27)
% Time in Target Range (70-180 mg/dL) - *Mean (SD)*	49% (21%)	62% (15%)
% Time >180 mg/dL - *Mean (SD)*	47% (24%)	35% (16%)
% Time >250 mg/dL – *Mean (SD)* [b]	19.8 (16.0%)	10.1% (8.6%)
% Time >300 mg/dL – *Mean (SD)* [b]	8.7% (9.3%)	3.5% (4.5%)
% Time <70 mg/dL – *Mean (SD)* [b]	3.6% (4.6%)	2.9% (3.5%)
% Time <54 mg/dL – *Mean (SD)* [b]	0.78% (1.56%)	0.50% (0.96%)
Coefficient of Variation – *Mean (SD)* [b]	37% (7%)	36% (5%)
HbA1c	N=113	N=110
Mean (SD)	7.8% (1.5%)	7.1% (1.0%)

[a]None of the T2D participants were using CGM at Enrollment.
[b]For metrics with a skewed distribution robust means using an M-estimator were calculated to down-weight outliers.

© 2023 Aleppo G et al. *JAMA Network Open.*

eTable 3. 6-Month Participant-Reported Questionnaire

	T1D (N=150) No. (%)	T2D (N=63) No. (%)
**Do you think that using a Continuous Glucose Monitor (CGM) helped you to manage your diabetes better?[a]** *n(%)*		
Yes	146 (>99%)	61 (100%)
No	1 (<1%)	0
**How do you look at the CGM data to help to manage your diabetes?** *n(%)*		
Real-time alerts and trends on CGM receiver or phone app	140 (93%)	58 (92%)
DreaMed Advisor Graphs and Reports that show your patterns over longer periods of time[b]	51 (34%)	30 (48%)
Dexcom Clarity Graphs and Reports that show your patterns over longer periods of time	110 (73%)	51 (81%)
I don't look at any of these	2 (1%)	0
**How often do you look at the CGM graphs that show your patterns over longer periods of time? These are the graphs like the Ambulatory Glucose Profile (AGP) that are available through DreaMed Advisor and Dexcom Clarity[a].** *n(%)*		
At least Weekly	61 (41%)	46 (75%)
At least Monthly	70 (48%)	13 (21%)
Less than Monthly	10 (7%)	1 (2%)
I don't look at the CGM graphs	6 (4%)	1 (2%)
**Did you change your eating habits based on what you saw on CGM?** *n(%)*		
Changed what food I ate	55 (52%)	47 (81%)
Changed how much of what I ate	62 (59%)	43 (74%)
Changed how often I ate	35 (33%)	24 (41%)
I found certain foods increased my blood sugars, so I reduced the amount, eliminated them from my diet, or decreased the frequency of how often I ate them	82 (78%)	52 (90%)
Other	13 (12%)	5 (9%)
Did not change any eating habits based on what I saw on CGM	42 (29%)	3 (5%)
**Have you discussed your CGM results with your personal health care provider who usually prescribes your insulin?[a]** *n(%)*		
Yes	103 (70%)	54 (89%)
No	44 (30%)	7 (11%)
**Working with the Virtual Clinic helped me better manage my diabetes.[a]** *n(%)*		
Strongly Agree	105 (71%)	52 (85%)
Agree	33 (22%)	9 (15%)
Neutral	4 (3%)	0
Disagree	3 (2%)	0
Strongly Disagree	2 (1%)	0
**If you had the option to continue to work with the Virtual Clinic, would you like to continue to work with them?[a]** *n(%)*		
Yes	132 (90%)	60 (98%)
No	15 (10%)	1 (2%)
**How has your physical activity level changed since you started using a CGM and working with the Virtual Clinic?[a]** *n(%)*		
More active	61 (41%)	31 (51%)
No Change	79 (54%)	28 (46%)
Less active	7 (5%)	2 (3%)

a – Three in the T1D group and 2 in the T2D did not answer the question.
b – Participants could view data through the Advisor Pro app

eTable 4. Participant-Reported Outcomes by Diabetes Type

T1D				
	**Baseline (N=149)**	**Month 3 (N=126)**	**Month 6 (N=116)**	**p-Value for change from Baseline to Month 6[a]**
**Patient Health Questionnaire-8 Mean Score** - *mean (SD)*	4.1 (4.1)	3.4 (3.7)	3.2 (4.0)	0.004
**Change from Baseline** - *mean (SD)*	NA	-0.3 (3.0)	-0.8 (2.9)	
**Diabetes Distress Scale Management Distress Subscale Mean Score** - *mean (SD)*	2.0 (0.8)	1.6 (0.6)	1.6 (0.6)	<0.001
**Change from Baseline** - *mean (SD)*	NA	-0.3 (0.6)	-0.3 (0.6)	
**Fear of Hypoglycemia Worry Subscale Mean Score** - *mean (SD)*	7.8 (5.2)	4.9 (5.0)	4.7 (4.5)	<0.001
**Change from Baseline** - *mean (SD)*	NA	-2.5 (4.1)	-2.9 (4.2)	
**T2D**				
	**Baseline (N=64)**	**Month 3 (N=55)**	**Month 6 (N=52)**	**p-Value for change from Baseline to Month 6**
**Patient Health Questionnaire-8 Mean Score** - *mean (SD)*	4.8 (4.5)	3.7 (4.6)	3.6 (5.0)	0.10
**Change from Baseline** - *mean (SD)*	NA	-1.3 (3.2)	-1.3 (5.4)	
**Diabetes Distress Scale Management Distress Subscale Mean Score** - *mean (SD)*	2.1 (0.9)	1.5 (0.6)	1.4 (0.6)	<0.001
**Change from Baseline** - *mean (SD)*	NA	-0.6 (0.8)	-0.8 (0.9)	
**Fear of Hypoglycemia Worry Subscale Mean Score** - *mean (SD)*	5.3 (5.8)	3.1 (4.0)	3.4 (5.3)	0.02
**Change from Baseline** - *mean (SD)*	NA	-2.6 (5.5)	-2.8 (6.2)	

[a] P-values were adjusted for multiple comparisons using the Two Stage Benjamini-Hochberg adaptive false discovery rate procedure.

© 2023 Aleppo G et al. *JAMA Network Open.*
