# Supplementary material for: Comprehensive Telehealth Model to Support Diabetes Self-Management
Source: JAMA Netw Open. 2023 Oct 4;6(10):e2336876. doi: 10.1001/jamanetworkopen.2023.36876 (PMC10551767; doi:10.1001/jamanetworkopen.2023.36876)
Supplement: Supplement 2. — Data Sharing Statement [file jamanetwopen-e2336876-s002.pdf]

Data Sharing Statement

Aleppo. Comprehensive Telehealth Model to Support Diabetes Self-Management. *JAMA Netw Open*. Published October 04, 2023. doi:10.1001/jamanetworkopen.2023.36876

Data
Data available: Yes
Data types: Deidentified participant data
How to access data: A documented, de-identified dataset may be provided to other researchers upon submitting a request to the corresponding author (rgal@jaeb.org) that describes the data requested and the purpose for its use.
When available: With publication

Supporting Documents
Document types: None

Additional Information
Who can access the data: A documented, de-identified dataset may be provided to other researchers upon submitting a request to the corresponding author that describes the data requested and the purpose for its use.
Types of analyses: Any purpose
Mechanisms of data availability: Without investigator support.
